# The PHES battery does not detect all cirrhotic patients with early neurological deficits, which are different in different patients

**DOI:** 10.1371/journal.pone.0171211

**Published:** 2017-02-01

**Authors:** Carla Giménez-Garzó, Juan José Garcés, Amparo Urios, Alba Mangas-Losada, Raquel García-García, Olga González-López, Remedios Giner-Durán, Desamparados Escudero-García, Miguel Angel Serra, Emilio Soria, Vicente Felipo, Carmina Montoliu

**Affiliations:** 1 Laboratorio de Neurobiología, Centro Investigación Príncipe Felipe de Valencia, Valencia, Spain; 2 IDAL, Intelligent Data Analysis Laboratory, Escuela Técnica Superior de Ingeniería, Valencia, Spain; 3 Fundación Investigación Hospital Clínico de Valencia. Instituto de Investigación Sanitaria-INCLIVA, Valencia, Spain; 4 Servicio de Digestivo, Hospital Arnau de Vilanova, Valencia, Spain; 5 Unidad de Digestivo, Hospital Clínico de Valencia, Departamento de Medicina, Universidad de Valencia, Valencia, Spain; 6 Departamento de Patología, Facultad de Medicina, Universidad de Valencia, Valencia, Spain; Taipei Veterans General Hospital, TAIWAN

## Abstract

**Background and aims:**

The psychometric hepatic encephalopathy score (PHES) is the “gold standard” for minimal hepatic encephalopathy (MHE) diagnosis. Some reports suggest that some cirrhotic patients “without” MHE according to PHES show neurological deficits and other reports that neurological alterations are not homogeneous in all cirrhotic patients. This work aimed to assess whether: 1) a relevant proportion of cirrhotic patients show neurological deficits not detected by PHES; 2) cirrhotic patients with mild neurological deficits are a homogeneous population or may be classified in sub-groups according to specific deficits.

**Methods:**

Cirrhotic patients “without” (n = 56) or “with” MHE (n = 41) according to PHES and controls (n = 52) performed psychometric tests assessing attention, concentration, mental processing speed, working memory and bimanual and visuomotor coordination. Heterogeneity of neurological alterations was analysed using Hierarchical Clustering Analysis.

**Results:**

PHES classified as “with” MHE 42% of patients. Around 40% of patients “without” MHE according to PHES fail two psychometric tests. Oral SDMT, d2, bimanual and visuo-motor coordination tests are failed by 54, 51, 51 and 43% of patients, respectively. The earliest neurological alterations are different for different patients. Hierarchical clustering analysis shows that patients “without” MHE according to PHES may be classified in clusters according to the tests failed. In some patients coordination impairment appear before cognitive impairment while in others concentration and attention deficits appear before.

**Conclusions:**

PHES is not sensitive enough to detect early neurological alterations in a relevant proportion of cirrhotic patients. Oral SDMT, d2 and bimanual and visuo-motor coordination tests are more sensitive. The earliest neurological alterations are different in different cirrhotic patients. These data also have relevant clinical implications. Patients classified as “without MHE” by PHES belonging to clusters 3 and 4 in our study have a high risk of suffering clinical complications, including overt HE and must be diagnosed and clinically followed.

## Introduction

Hepatic encephalopathy (HE) is a complex neuropsychiatric syndrome present in patients with chronic liver diseases that leads to alterations in personality, sleep, cognitive function, motor activity and coordination and level of consciousness and may lead to coma and death [1–2]. Around 33%-50% of cirrhotic patients without clinical symptoms of HE show minimal hepatic encephalopathy (MHE), which can be unveiled using psychometric tests or neurophysiological analysis [2–5]. MHE reduces quality of life and life span and is associated with increased risk of work, driving, and home accidents and predisposes to clinical HE [6–11]. Patients with MHE show mild cognitive impairment, attention deficits [12–17] and impaired visuo-motor and bimanual coordination [18–19].

The existence of MHE was realized many years ago and was referred as subclinical HE or other names. However, the psychometric tests performed in different settings were so heterogeneous that it was difficult to properly compare different studies and characterize the neurological alterations in MHE. To solve these problems a consensus was reached to use the psychometric hepatic encephalopathy score (PHES) as the “gold standard” for diagnosis of MHE [2]. PHES is a battery of five psychometric tests assessing different functions including attention and fine motor coordination. The PHES battery has been very useful to homogenize the assessment of MHE worldwide. EASL/AASLD guidelines on HE reflected the difficulties of the different tests used for the diagnosis of MHE or Covert HE (CHE) and a poor correlation between them, which reveals the lack of an operative guidance on how to combine test results for diagnosing MHE [20].

Recent studies have shown that cirrhotic patients who are classified as “without” MHE using the PHES battery may already present some neurological deficits. Butz et al [21] showed that ataxia, tremor, and slowing of finger movements are early markers for cerebral dysfunction in at least a subgroup of cirrhotic patients even prior to alterations in performance in the PHES become detectable. Felipo et al [18,19] showed that some cirrhotic patients classified as “without MHE” by the PHES already showed deficits in attention tests (Stroop) and in bimanual coordination. These data indicate that some mild neurological alterations are not detected using the PHES but can be detected using more sensitive tests [18–19, 21]. This suggests that, although the PHES has been very useful to homogenize the assessment of neurological alterations in cirrhotic patients, it is not sensitive enough to detect some mild alterations. This would mean that some patients with neurological impairment would not be properly diagnosed using the PHES.

On the other hand, some studies also suggest that the neurological alterations are not homogeneous in all cirrhotic patients. The motor alterations reported by Butz et al [21] seem to occur only in a subgroup of cirrhotic patients “without MHE” according to PHES. Felipo et al [19] showed that some patients are classified as with MHE by the PHES mainly due attention deficits and others mainly due to motor coordination deficits. This suggests that cirrhotic patients could be a heterogeneous population: some of them could develop earlier cognitive deficits and others motor alterations.

The aims of this study were to assess whether:

1. there is a relevant proportion of cirrhotic patients showing neurological deficits that are not detected by the PHES.
2. cirrhotic patients with mild neurological deficits behave as an homogeneous population or may be classified in sub-groups according to their specific deficits.

To reach these aims we performed in cirrhotic patients classified as “without” or “with” MHE according to PHES and in control subjects a systematic analysis of different neurological functions using psychometric tests comprising a total of 20 sub-tests assessing more sensitively attention, concentration, mental processing speed, working memory and bimanual and visuomotor coordination. We tested if some patients classified as “without” MHE by the PHES show relevant deficits in any of the above tests.

To assess the homogeneity or heterogeneity of the earliest neurological alterations in cirrhotic patients we performed Exploratory Data Analysis and Hierarchical Clustering to arrange the subjects according to the similarity of their performances in 12 tests using “Jaccard” dissimilarity measures. The results of the hierarchical clustering are presented in a dendrogram.

## Materials and methods

### Patients and controls

Ninety-seven patients with cirrhosis and 52 controls were enrolled in the study after signing a written informed consent. The diagnosis of cirrhosis was based on clinical, biochemical, and ultrasonographic data. Exclusion criteria were overt HE or history of overt HE, recent (<6 months) alcohol intake, infection, recent (<6 weeks) gastrointestinal bleeding, or use of antibiotics or drugs affecting cognitive function, shunt surgery or transjugular intrahepatic portosystemic shunt, electrolyte imbalance, renal impairment (serum creatinine >1.5 mg/dL), or hepatocellular carcinoma. Controls were included in the study once liver disease was discarded by clinical, analytical, and serologic tests.

Study protocols were approved by the Scientific and Ethical Committees of Hospitals Clínico and Arnau de Vilanova, Valencia, Spain and were in accordance with the ethical guidelines of the Helsinki Declaration [22]. After performing the psychometric tests, patients were classified as without MHE (56 patients) or with MHE (41 patients) according to PHES (see below). The study includes therefore three groups: 1) control subjects 2) patients without MHE and 3) patients with MHE (MHE). The composition of the groups, age, and aetiology of the disease are given in Table 1.

**Table 1:** Composition of the different groups and etiology of liver disease.

	CONTROLS	PATIENTS without MHE	PATIENTS with MHE
**Total individuals**	52	56	41
**Gender (M/F)**	27/25	48/8	25/16
**Age** *	56 ± 8	59 ± 10	64 ± 10
Educational level (years)*	11 ± 0.5	11 ± 0.5	10 ± 0.4
**Alcohol**	-----	28	15
**HBV/ HCV/HBV+HCV**	-----	1/21/1	3/13/0
**Alcohol +HBV/ HCV**	-----	2/1	1/4
**Others**		2	5
**Ascites**	-----	6	6
**Child Pugh A/B/C**	-----	40/16/0	25/12/4
MELD*	-----	9 ± 3	10 ± 5

*Values are expressed as mean ± SD. MHE, minimal hepatic encephalopathy; HBV, hepatitis B virus; HCV, hepatitis C virus. MELD, model end stage liver disease. The Child Pugh Score is derived from a score of 1–3 given for severity of ascites, hepatic encephalopathy, INR, albumin and bilirubin. The higher the score is, the more severe the liver disease.

Clinically relevant outcomes were assessed by revising the patient’s medical records from the date of inclusion in the study to 12–48 months follow-up. The number and percentage of clinical complications were determined for each cluster of patients. The main outcomes were death and decompensations due to liver disease: overt HE, ascites, variceal bleed, spontaneous bacterial peritonitis, hepatocellular carcinoma…

### Neuropsychological assessment

#### Diagnosis of MHE

Psychometric Hepatic Encephalopathy Score (PHES) [2] was used for diagnosis of MHE. PHES comprises a battery of 5 psychometric tests: digit symbol test (DST), number connection test A (NCT-A), number connection test B (NCT-B), serial dotting test (SD), and the line tracing test (LTT) [23]. The global PHES score and from each test were calculated adjusting for age and education level by Spanish normality tables (www.redeh.org). Patients were considered as having MHE when the score was ≤ −4 points [23].

#### Critical Flicker Frequency (CFF)

The CFF was measured as in [24].

#### Stroop test

Selective attention was assessed with a colour-word version of Stroop test, by performing sequentially the congruent, neutral and incongruent tasks, 45 seconds per task, as in [18]. The number of items correctly named was adjusted by age according to Spanish normality tables.

#### Bimanual and visuo-motor coordination tests

Bimanual and visuo-motor coordination tests were performed as in [18] and time in minutes was recorded for each test.

#### d2 test

This test evaluates selective/sustained attention and mental concentration and provides scores reflecting three components of attentional behaviour: speed or amount of work done in a given time; accuracy of such work and relationship between speed and accuracy [25,26].

The d2 test is a one-page paper-and-pencil cancellation test consisting of 14 rows (trials), each with 47 interspersed “p” and “d” characters [26]. The characters have one to four dashes presented individually or in pairs above and/or below each letter. The target symbol is a “d” with two dashes (“d2”), regardless of whether the dashes appear above, below, or one above and one below the “d”. A “p” or a “d” with more or less than two dashes are distracters. The participant’s task is to cancel as many target symbols as possible in 20 s/trial. The test provides the following parameters: Total number of characters processed (TR); omission errors (O) (number of target symbols not cancelled); commission errors (C) (number of non-target symbols cancelled); total errors (O_C) (sum of omission and commission errors); total correctly processed (TOT) (total characters processed minus total errors made); (CON) concentration performance (number of correctly minus incorrectly cancelled items); right answers (TA) (number of characters correctly cancelled), and fluctuation rate (VAR) (maximum total items processed in a trial minus minimum total items processed in a trial).

#### Symbol Digit Modalities Test (Oral SDMT)

This test consists of a series of nine symbols in which every symbol is paired with a single digit, labeled 1–9. In the test page a sequence of symbols is presented, and patients have to tell the correct digit associated to every symbol in 90 seconds [27]. Number of total item, correct pairings and errors are registered.

#### Digit span

This test evaluates immediate and working memory (Wechsler Adult Intelligence Scale, WAIS) [28]. It consists of two parts: ‘digits forward’, and ‘digits backward’ that were performed as in [29].

#### Letter-number sequencing

This test measures working memory, having more working memory load than digit span [30]. It consists of three-series blocks containing mixed letters and numbers, and the number of elements increases as the test progresses. After hearing a series, the subject had to sort the items, saying the numbers in ascending order and then the letters alphabetically arranged. The test continued until the subject failed three series of a block. Total correct answers were registered.

### Statistical analysis for individual tests

Values are given as mean±SEM unless otherwise specified. Results were analyzed by one-way ANOVA followed by post-hoc Bonferroni’s multiple comparison test. Variables that were not previously age-adjusted (bimanual and visuo-motor coordination tests) were analysed using univariate analysis of covariance (ANCOVA) with age included as covariate, followed by post-hoc Bonferroni. Analyses of contingency tables of clinical outcomes in the different clusters were performed by Fisher’s exact test. For coordination tests (bimanual and visuo-motor coordination tests) statistical analyses were performed by including the age as a covariate. For the other tests the parameters measured for each participant were adjusted by age according to test manual and Spanish normality tables. Analyses were performed using GraphPad Prism version 6.0 and SPSS version 19.0 (SPSS Inc, Chicago, USA) and two-sided P values <0.05 were considered significant.

### Integrated analysis of performance in the different tests and of heterogeneity of the groups of patients

This analysis was done using Exploratory Data Analysis (EDA) followed by more advanced techniques: Hierarchical Clustering and Dendrogram. The score of each test was translated into a binary format (1 means failed test and 0 passed test). The general fail criterion was to obtain a score equal or lower than the mean minus 2 times the standard deviation of the control group. For visual and bimanual coordination tests, the criterion was to obtain a score equal or higher than the mean plus 2 times the standard deviation of the control group, as in [31]. NA (*Not Available*) value was used for not performed tests (11 patients with MHE and 13 without MHE did not performed a few (1–4) tests).

#### Exploratory Data Analysis (EDA)

EDA employs a variety of techniques to maximize insight into a data set, uncover underlying structure, extract relevant variables and detect outliers and anomalies. It allows testing underlying assumptions, develop parsimonious models and determine optimal factor settings [32]. EDA was used for calculation of the main statistics values and proportions, including means, variances, standard deviations, number of tests failed, number of NA’s and percentage of tests failed for each subject and group, number and percentage of fails by tests and groups, quantity of *n* or more fails by groups, etc.

#### Hierarchical clustering and dendrogram

Items were arranged in a hierarchy based on the similarity between them [33]. The hierarchy has different levels representing particular grouping of the data into disjoint clusters. This provides clusters including subsets of data that are more closely related between them than subjects assigned to a different cluster [33]. The results of the hierarchical clustering calculation have been visualized in a dendrogram, a tree-structured graph [34]. For each subject studied, a list of failed tests was created. Each subject is an item for the hierarchical clustering. The distance between items has been computed using “Jaccard” dissimilarity measure: the number of items which occur in both elements divided by the total number of items in the elements [35]. The distance value is between 1 (very different) and 0 (no distance = no difference between elements). A vertical dendrogram has been created, and the number identifying each patient has been coloured according to their classification by the PHES: control (black), without MHE (red) or with MHE (green).

## Results

**Fig 1:**
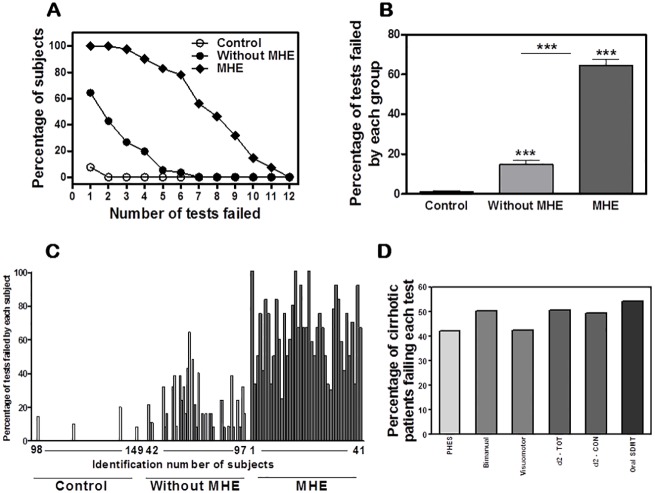
Patients “without” MHE according to PHES show impaired performance in some psychometric tests. The tests were performed by patients classified as “without” or “with” MHE according to PHES and by controls. (A) Percentage of individuals of each group that fail the indicated number of tests or more. (B) Percentage of tests failed by each group (mean±SD). (C) Each bar represents the percentage of tests failed by each individual subject. (D) Percentage of total cirrhotic patients (“without” or “with” MHE according to PHES) failing the indicated tests: Oral SDMT test, total items; d2 test, TOT: total correctly processed; bimanual coordination; concentration, measured with the d2 test and in visuo-motor coordination.

The different tests and subtests performed provide, in addition to the PHES scores, 20 additional scores for parameters measuring different aspects of cognitive and motor function. These scores for cirrhotic patients classified as “without” or “with” MHE according to PHES and of controls are given in Table 2 (PHES scores) and Table 3 (all other scores). After applying Exploratory Data Analysis, the scores for PHES, critical flicker frequency and for 10 of these parameters (named tests for simplicity from now on) were selected to compare performance of patients “without” or “with” MHE and controls. The results are summarized in Fig 1.

**Table 2:** Performance in the PHES and in each of its tests.

TEST	CONTROLS	PATIENTS without MHE *P* vs. Control	PATIENTSwith MHE *P* vs. control	PATIENTS withMHE *P* vs. without MHE	Global ANOVA*P* Values
**CFF (Hz)**	43.2 ± 0.04	41.± 0.4 p<0.01	38 ± 0.5 p<0.001	**p<0.001**	**<0.001**
**PHES Global score** *	0.04 ± 0.13	-0.52 ± 0.15 p<0.05	-6.8 ± 0.4 p<0.001	**p<0.001**	**<0.001**
(DST) Digit Symbol Test*	0.12 ± 0.05	-0.16 ± 0.05 p<0.01	-0.68 ± 0.09 p<0.001	**p<0.001**	**<0.001**
NCT-A*	0.0 ± 0.08	0.14 ± 0.07	-1.17 ± 0.17 p<0.001	**p<0.001**	**<0.001**
NCT-B*	0.04 ± 0.06	-0.20 ± 0.08	-1.95 ± 0.19 p<0.001	**p<0.001**	**<0.001**
(SD) Serial Dotting Test*	-0.04 ± 0.03	-0.13 ± 0.05	-0.85 ± 0.16 p<0.001	**p<0.001**	**<0.001**
(LTT) Line Tracing Test*	-0.06 ± 0.05	-0.11 ± 0.07	-2.15 ± 0.14 p<0.001	**p<0.001**	**<0.001**

Values are expressed as mean ± SEM.

*Games-Howell test was done due to non-homogeneity of variances. MHE, minimal hepatic encephalopathy; CFF, Critical Flicker Frequency; PHES, Psychometric Hepatic Encephalopathy Score; NCT-A, NCT-B, Number Connection Test A and B, respectively.

**Table 3:** Performance in psychometric tests.

TEST	CONTROLS	PATIENTS without MHE *P* vs. Control	PATIENTS with MHE *P* vs. control	PATIENTS with MHE *P* vs. without MHE	Global ANOVA*P* Values
**Bimanual coordination (min)**	1.76 ± 0.03	2.00 ± 0.04 p<0.05	2.73 ± 0.10 p<0.001	**p<0.001**	**<0.001**
**Visuo-motor coordination (min)**	2.26 ± 0.05	2.55 ± 0.05 p<0.05	3.40 ± 0.10 p<0.001	**p<0.001**	**<0.001**
**d2 Test**					
***TR_****Values*	430 ± 11	360 ± 11 p<0.001	255± 11 p<0.001	**p<0.001**	**<0.001**
***TOT_*** *Values*	414 ± 11	344 ± 11 p<0.001	230 ± 11 p<0.001	**p<0.001**	**<0.001**
***CON_*** *Values*	158 ± 5	135 ± 5 p<0.05	82 ± 6 p<0.001	**p<0.001**	**<0.001**
***VAR_*** *Values*	13 ± 1	12 ± 0.5	14 ± 1	**ns**	**ns**
***TA_*** *Values*	161 ± 5	139 ± 4 p = 0.008	88 ± 5 p<0.001	**<0.001**	**<0.001**
***O_*** *Values* *	15 ± 2	12 ± 1	22 ± 3	**p<0.05**	**0.008**
***C_*** *Values* *	0.7 ± 0.2	2.6 ± 0.8	5 ± 1 p = 0.001	**ns**	**0.003**
***O_C_*** *Values* *	16 ± 2	15 ± 2	27 ± 4 p<0.05	**p<0.05**	**0.002**
**STROOP Test**					
***Congruent Task (Number of words)***	112 ± 2	104 ± 2	82 ± 3 p<0.001	**p<0.001**	**<0.001**
***Neutral Task (Number of colours)***	81 ± 2	75 ± 2 p<0.05	59 ± 2 p<0.001	**p<0.001**	**<0.001**
***Incongruent Task (Number of items)***	48 ± 1	40 ± 1 p = 0.001	32 ± 1 p<0.001	**p<0.001**	**<0.001**
**Oral SDMT Test**					
***Total items***	53.6 ± 1.7	41 ± 1 p<0.001	27 ± 2 p<0.001	**p<0.001**	**<0.001**
***Correct pairings***	53 ± 2	40.6 ± 1.3 p<0.001	26 ± 2 p<0.001	**p<0.001**	**<0.001**
***Errors (% over total items)***	1.4 ± 0.5	2.4 ± 0.5	5 ± 1 p<0.01	**p<0.05**	**<0.01**
**DIGIT SPAN Test**					
***Digits forward (right answers)***	9 ± 0.4	8 ± 0.3 p<0.05	7 ± 0.3<0.001	**ns**	**<0.001**
Digits backward (right answers)*	6.3 ± 0.4	5 ± 0.2 p<0.01	3.5 ± 0.2 p<0.001	**p<0.001**	**<0.001**
Digitos Total score*	16 ± 1	12 ± 0.4 p<0.01	10.4 ± 0.4 p<0.001	**p<0.01**	**<0.001**
***Letter-Number Sequencing test (right answers)***	10 ± 0.5	7 ± 0.4 p<0.001	4.6 ± 0.4 p<0.001	**p<0.001**	**<0.001**

Bimanual and Visuo-motor coordination tests: score in minutes; Stroop test: Congruent task: number of words read in 45 seconds; Neutral task: number of colours read in 45 seconds; Incongruent task: number of items completed in 45 seconds. Oral SDMT, Symbol digit modalities test (oral version). TR, Total number of characters processed; TOT, Total correctly processed; CON, Concentration performance; VAR, fluctuation rate; TA, Total right answers; O, errors of omission; C, errors of commission; O_C, Total errors. Differences between groups were analyzed using one-way ANOVA followed by post-hoc Bonferroni, with the exception of parameters labelled with *, in which Games-Howell test was done due to non-homogeneity of variances. For Bimanual and Visuo-motor coordination tests, univariate analysis of covariance (ANCOVA) was performed, with age included as covariate, followed by post-hoc Bonferroni. For the other tests the parameters measured for each participant were adjusted by age according to test manual and Spanish normality tables.

Fig 1A shows that controls perform correctly essentially all tests. However, patients “without” MHE already show deficits in a significant number of tests. Around 40% of them show deficits in at least 2 of the 12 tests. Patients “with” MHE show deficits in many of the tests and 50% of them have deficits in at least 7 of them.

Patients “with” MHE failed 64±3% of the tests, patients “without” MHE 15±2% and controls 1±0.5% of the tests (Fig 1B). The percentage of tests failed by each individual is shown in Fig 1C.

The percentage of cirrhotic patients showing deficits in each of the 12 tests are shown in Fig 1D. Forty-two percent of cirrhotic patients show deficits in the PHES and are therefore classified as “with” MHE. The percentage of patients that fail the test is higher for 5 tests than for the PHES.

The highest proportion of deficits occurs for the Oral SDMT test (total items), which is failed by 54% of the patients. The d2 test (TOT: total correctly processed) and bimanual coordination are impaired in 51% of the patients. Concentration, measured with the d2 test is impaired in 49% of the patients and visuo-motor coordination in 43% of them (Fig 1D).

**Fig 2:**
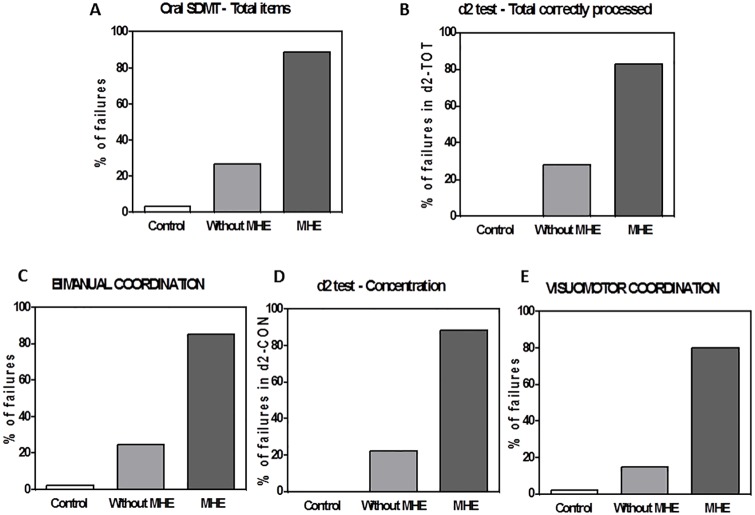
Percentage of individuals of each group showing impaired performance in each test. The tests were performed by patients classified as “without” or “with” MHE according to PHES and by controls. The percentage of individuals of each group showing impaired performance in Oral SDMT test, total items (A); d2 test, TOT: total correctly processed (B); bimanual coordination (C); concentration, measured with the d2 test (D) and in visuo-motor coordination (E), are given. The failure criterion was to obtain a score equal or lower than the mean minus 2 times the standard deviation of the control group in A, B and D and equal or higher than the mean plus 2 times the standard deviation of the control group in C and E.

The percentage of subjects of each group failing these tests is shown in Fig 2. For patients “without” MHE, 27%, 28%, 26%, 22% and 15% show deficits in the Oral SDMT test, d2 test (total correctly processed), bimanual coordination, concentration in d2 test and visuo-motor coordination, respectively. The corresponding percentages in patients “with” MHE are 89%, 83%, 85%, 89% and 80%, respectively (Fig 2).

**Fig 3:**
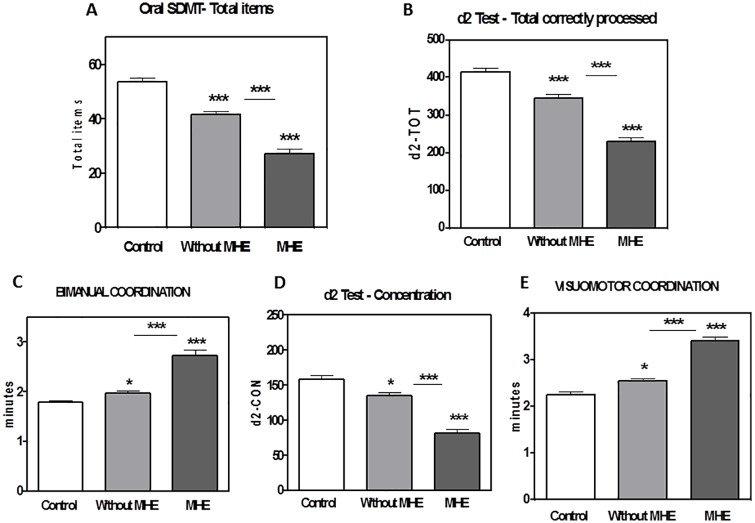
Patients “without” MHE according to PHES show impaired performance in Oral SDMT test, d2 test and bimanual and visuo-motor coordination. The mean scores of each group in Oral SDMT test, total items (A); d2 test, TOT: total correctly processed (B); bimanual coordination (C); concentration, measured with the d2 test (D) and in visuo-motor coordination (E), are given. Values are the mean±SEM of 56 patients classified as “without”, 41 “with” MHE according to PHES and 52 controls. Values significantly different from controls are indicates by asterisks: *p<0.05; ***p<0.001.

The mean scores of each group in these five tests are given in Fig 3A: Oral SDMT test (total items), Fig 3B: d2 test (TOT: total correctly processed), Fig 3C: bimanual coordination, Fig 3D: concentration, measured with the d2 test, and Fig 3E: visuo-motor coordination. Patients “without” MHE perform significantly worse than controls in all these 5 tests. These data support that these tests could be more sensitive than the PHES to detect neurological impairment in cirrhotic patients.

**Fig 4:**
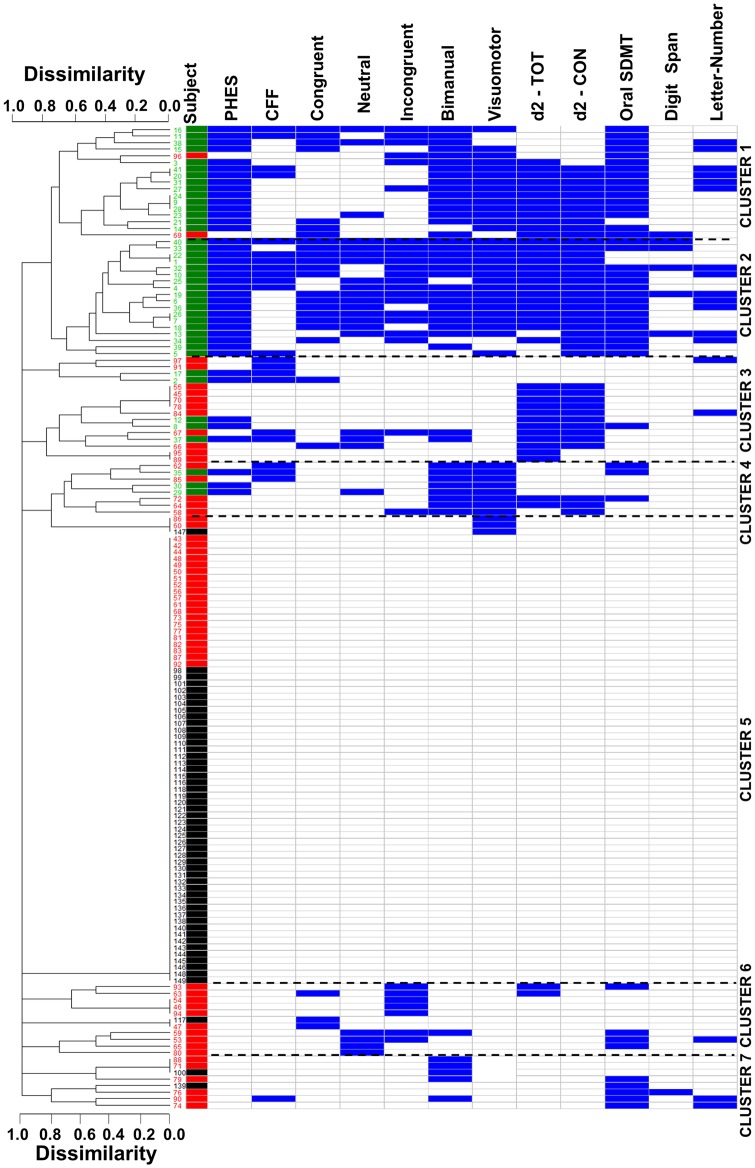
Dendrogram showing the clustering of all individuals in main groups and sub-groups. All subjects included in the study have been arranged according to the similarity-dissimilarity of their performance in the combination of tests indicated using hierarchical clustering analysis. This analysis provides clusters including subjects more closely related between them than subjects assigned to a different cluster. These clusters are visualized in the dendrogram shown. The first column shows the number identifying each subject, which have been coloured according to their classification by the PHES: control (black), without MHE (red) or with MHE (green). These colours are repeated in the second column to facilitate its identification. The tests failed by each individual are indicated by blue colour in the corresponding box. The following tests have been included: PHES, critical flicker frequency (CFF), the congruent, neutral and incongruent tasks of the Stroop test, Bimanual and visuo-motor coordination, d2 test, TOT: total correctly processed and d2-CON: concentration, measured with the d2 test; Oral SDMT test, Digit Span and Letter-number test.

Concerning CFF, the concordance with PHES was 68%. Of the 97 patients, 50 were classified as without MHE and 16 as with MHE both by PHES and CFF. Six patients were classified as with MHE by CFF but not by PHES and 25 were calssified as with MHE by PHES but not by CFF (Fig 4).

To assess if cirrhotic patients with early neurological deficits behave as a homogeneous population or may be classified in sub-groups according to their specific deficits we performed a Hierarchical Clustering Analysis which results are presented in the dendrogram shown in Fig 4.

The dendrogram shows several clusters of individuals. The clearer cluster corresponds to the control group (black colour in the number labels) with no tests failed (cluster 5). Essentially all controls appear together in the dendrogram indicating high homogeneity of the group.

Patients with MHE (green colour) also form a clear cluster in the upper part of the dendrogram. They come from a principal branch with several ramifications meaning that there are small differences between them. There are two main branches or sub-groups. The bottom one is composed by patients “with” MHE who failed mainly in Digit Span and Stroop tests (cluster 2) while patients in the upper one did not fail in these tests (cluster 1).

Patients without MHE (red colour) are more disseminated and the dendrogram suggests several sub-groups. The first sub-group is in the same branch than control subjects and is composed of patients “without” MHE who did not fail any test (cluster 5). The second and third sub-groups are located between the first one and the cluster of patients “with” MHE (green). The second sub-group is composed essentially by patients “without” MHE who failed in d2 tests (cluster 3) while those in the third sub-group failed mainly in visuo-motor and bimanual coordination tests (cluster 4).

Two additional sub-groups of patients “without” MHE are at the bottom of the dendrogram, far from other sub-groups, indicating significant dissimilarities with them. This fourth sub-group failed mainly bimanual coordination and Oral SDMT tests (cluster 7) and the fifth sub-group had a combination of fails in the Stroop and Oral SDMT tests (cluster 6) (Fig 4).

### Follow-up of clinically relevant outcomes

**Fig 5:**
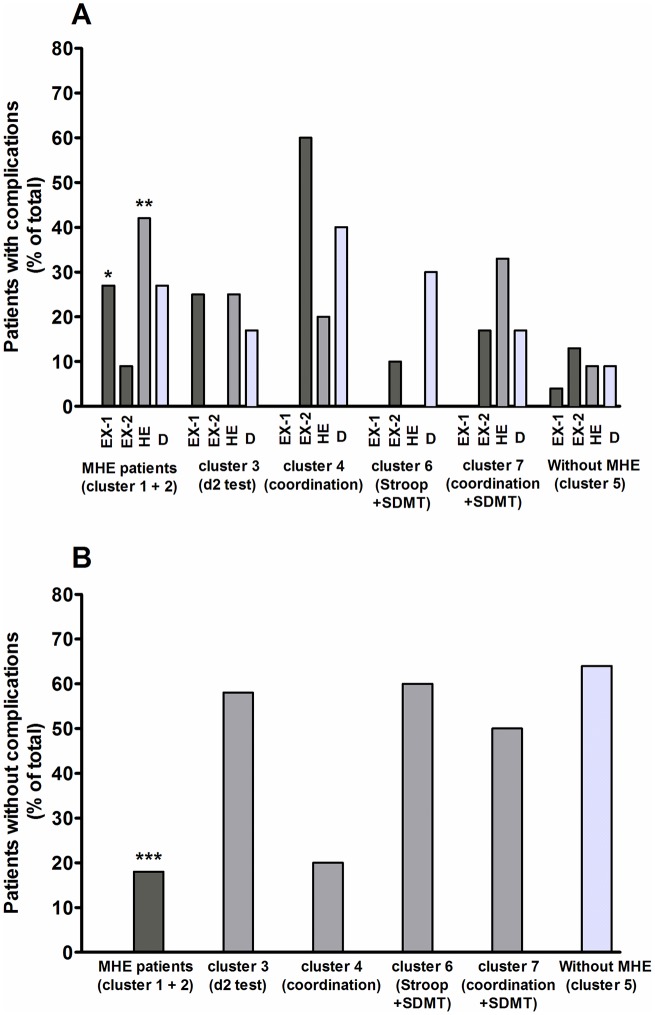
Follow-up of clinically relevant outcomes in the different clusters of patients. (A) Percentage of patients with complications in each group. Different clinical complications are expressed as: EX-1, exitus by causes related to liver disease; EX-2, exitus by causes non-related to liver disease; HE, overt hepatic encephalopathy; D, other complications (ascites, variceal bleed, hepatocellular carcinoma, spontaneous bacterial peritonitis…). (B) Percentage of patients without complications in each group. In clusters 3 and 4 only patients without MHE (according to PHES score) are shown. Tests failed by patients in each group are shown in parentheses. Differences of all clusters were performed by Fisher’s exact test by comparing with cluster 5 (patients without MHE with no failed tests). *p<0.05; **p<0.01; ***p<0.001.

To assess whether patients missed by the PHES but showing some neurological alteration (clusters 3, 4, 6 and 7) have a higher progression to overt HE or some other clinically relevant outcome, we followed subsequent complications of cirrhosis and mortality in the different clusters of patients. These results are shown in Fig 5 and Table 4.

**Table 4:** Complications and mortality of patients from different clusters studied.

	CLUSTER 1 n = 17	CLUSTER 2 n = 18	CLUSTER 3 n = 16	CLUSTER 4 n = 8	CLUSTER 5 n = 22	CLUSTER 6 n = 10	CLUSTER 7 n = 6
Without MHE	2	0	12	5	22	10	6
With MHE	15	18	4	3	0	0	0
**Without MHE (n)**	2	0	12	5	22	10	6
Complications n (%)	0 (0)	-	5 (42)	3 (60)	4 (18)	3 (30)	3 (50)
HE	0 (0)	-	3 (25)	1 (20)	2 (9)	0 (0)	2 (33)
Other (Ascites, variceal bleed, infections, HCC)	0 (0)	-	2 (17)	2 (40)	2 (9)	3 (30)	1 (17)
Unknown	-	-	-	1 (20)	-	-	-
Without complications	2 (100)	-	7 (58)	1 (20)	14 (64)	6 (60)	3 (50)
Death n (%)	0 (0)	-	3 (25)	3 (60) (p = 0.09)	4 (18)	1 (10)	1 (17)
**With MHE (n)**	15	18	4	3	0	0	0
Complications n (%)	**9 (60)** *	14 (78)***	2 (50)	1 (33)	-	-	-
HE	5 (33) (p = 0.09)	9 (50)**	1 (25)	0	-	-	-
Other (Ascites, variceal bleed, infections, HCC)	4 (27)	5 (28)	1 (25)	1 (33)	-	-	-
Unknown	2 (13)	2 (11)	-	-	-	-	-
Without complications	4 (27)	2 (11)	1 (25)	2 (67)	-	-	-
Death n (%)	5 (33) (p = 0.09)	7 (39%)	1 (25)	0	-	-	-

HE, Hepatic encephalopathy; HCC, hepatocellular carcinoma; n, number of subjects. Data were analysed comparing all groups with Cluster 5 (patients without MHE with no failed tests) by Fisher’s exact test. P values are referred to Cluster 5:

*p<0.05;

**p<0.01;

***p<0.001.

Only 18% of patients without MHE showing no impairment in any test (cluster 5) presented complications in the follow-up study: 9% developed overt HE and 9% suffered other complications. One patient (4% of total) died by causes related to cirrhosis (Fig 5A).

As expected, more patients with MHE according to PHES, (clusters 1 and 2), presented complications (70%, p = 0.0002): 42% (p = 0.005) developed overt HE and 27% other complications. Nine patients (27% of total, p = 0.04) died by causes related to cirrhosis (Fig 5A).

Patients without MHE according to PHES but showing some neurological alteration in the other tests also show more complications than patients in cluster 5. This was more evident for patients in cluster 4, 60% of patients “without MHE” presented complications, 20% had HE and 40% other complications. In cluster 3, 42% of patients presented clinical complications, 25% had HE and 17% other complications. Three patients (25%) died by causes related to cirrhosis (Fig 5A).

Patients “without MHE” in clusters 6 and 7 also suffered more complications than those in cluster 5, with an incidence of complications of 30% and 50%, respectively. Two of the six (33%) patients in cluster 7 developed overt HE (Fig 5A).

Sixty-four percent of patients in cluster 5 did not develop any complication in the follow-up. In patients with MHE according to PHES (clusters 1–2) this was reduced to 18% (p = 0.0002) and in cluster 4 to 20% (Fig 5B). In clusters 3, 6 and 7 the percentages of patients without complications were 58%, 50% and 60%, respectively (Fig 5B).

These data show that patients in cluster 3 and, especially, those in cluster 4, show a high risk of suffering clinical complications, including overt HE.

## Discussion

This report provides the following relevant findings:

1. The PHES battery is not sensitive enough to detect early neurological alterations in a relevant proportion of cirrhotic patients. Around 40% of patients classified as “without” MHE by the PHES fail at least 2 of the psychometric tests performed.
2. Several psychometric tests: Oral SDMT, the d2 test (both total correctly processed and concentration) and bimanual and visuo-motor coordination tests are more sensitive than PHES in detecting early neurologic impairment in cirrhotic patients.
3. The earliest neurological alterations are not the same for all cirrhotic patients. In a sub-group of patients alterations in coordination appear before mild cognitive impairment while in another sub-group concentration and attention deficits appear before coordination impairment. This is clearly illustrated by the classification of patients “without” MHE according to PHES in different clusters or sub-groups in the dendrogram according to the types of tests failed.

Although the PHES has been very useful to homogenize assessment of MHE around the world, as indicated in the Introduction some recent reports suggest that cirrhotic patients classified as “without” MHE by the PHES may present neurological deficits which can be detected using more sensitive tests [18–19, 21].

We have now systematically assessed and analysed this possibility by performing a battery of sensitive tests and found that more than 40% of patients classified as “without” MHE by the PHES fail at least 2 of the psychometric tests performed. This indicates that a relevant proportion of cirrhotic patients with mild neurological impairment are not properly diagnosed using the PHES. This would delay its treatment and affect quality of life.

MHE has a significant impact on a patient's quality of life, driving performance, increased hospitalizations and death. Given its burden on patients, care takers, and health care systems, early diagnosis and management are imperative [36]. Treatment of MHE would improve quality of life, prevent progression and reduce societal costs by reducing the number of motor vehicle accidents [37]. Proper and sensitive diagnosis of MHE at the earliest possible stages is therefore necessary. The data reported show that PHES is not sensitive enough and more sensitive tests should be used to allow early diagnosis and treatment of MHE.

Moreover, as discussed below, the earliest neurological alterations are different in different patients. This means that using a single sensitive test is not enough and a combination of tests should be used. For example, 54% of patients fail in the Oral SDMT test (total items) and 51% in bimanual coordination. However, 68% of patients fail in one or the other. Therefore performing both tests would detect MHE more sensitively. Taking into account that PHES only detects MHE in 42% of the patients, the combination of Oral SDMT test and bimanual coordination test would increase by 62% de number of patients diagnosed for MHE. This would allow earliest management.

A few previous reports suggest that the earliest neurological alterations are not homogeneous in all cirrhotic patients. Butz et al [21] reported motor alterations only in a subgroup of cirrhotic patients “without MHE” according to PHES. Felipo et al [19] showed that some patients are classified as “with” MHE by the PHES mainly due attention deficits and others mainly due to motor coordination deficits. Montagnese et al [38] also suggested recently that “covert” HE (the combination of MHE and grade I HE) is a heterogeneous entity.

We have now used hierarchical clustering analysis to rigorously assess if cirrhotic patients are a heterogeneous population concerning their earliest neurological alterations. This analysis shows that patients classified as “without” MHE according to PHES may be classified in several sub-groups: one (cluster 5 in dendrogram) behaves as controls, do not show neurological alterations and do not fail in any test. A second sub-group fails d2 tests (cluster 3) and a third one (cluster 4) fails mainly coordination tests. A fourth sub-group (cluster 6) fails mainly in the Stroop test and a fifth sub-group (cluster 7) in a combination of bimanual coordination and oral SDMT.

These second to fifth sub-groups of patients, who are classified as “without” MHE by the PHES, would represent the mildest forms of MHE, presenting the earliest neurological alterations. It can be seen that these alterations are different in each sub-group, supporting that the earliest neurological alterations are heterogeneous, are not the same in all cirrhotic patients.

The neurological functions affected in different sub-groups are modulated by different mechanisms, involving different neurotransmitter systems in different brain areas. For example, cerebellum is critical in modulation of bimanual and visuo-motor coordination [39–41] and GABA is the main neurotransmitter modulating motor coordination [42–43]. In contrast, attention and executive function are largely mediated by prefrontal cortex and modulated by dopaminergic, noradrenergic, serotonergic, and cholinergic neurotransmission [44]. It is likely that in some patients GABAergic neurotransmission in cerebellum is affected earlier leading to motor coordination impairment while in other patients cholinergic neurotransmission in cortex is affected earlier leading first to attention and concentration deficits.

The dendrogam also shows that patients “with” MHE according to PHES show alterations both in coordination, concentration and attention (clusters 1 and 2). This suggests that independently of which are the initial alterations, all these cerebral functions are finally altered in cirrhotic patients.

A lack of concordance in the classification of cirrhotic patients as with or without MHE by different tools or tests has been already reported, e.g. CFF vs PHES, PHES vs. CRT, PHES vs ICT [20]. The results reported here help to clarify the reasons for this lack of concordance. We clearly show that patients with liver cirrhosis are a heterogeneous population concerning the earliest types of neurological alterations they develop. Some patients develop first attention deficits (cluster 3) while other develop first motor coordination alterations (cluster 4). These patients are classified by the PHES as without MHE and would be classified or not as with MHE depending on the test performed, the d2 test will classify patients in cluster 3 as with MHE and patients in cluster 4 as without MHE. However, the classification would be the opposite using visuo-motor or bimanual coordination tests, patients in cluster 3 will be classified as without MHE and patients in cluster 4 as with MHE. Only the combination of d2 and visuo-motor / bimanual coordination tests would detect all patients in clusters 3 and 4 with some neurological deficit.

Although performing a single test to detect MHE would be more convenient in clinical practice, the heterogeneity of the earliest neurological alterations in different individual patients precludes this approach, which will leave undiagnosed many patients with mild neurological alterations. A good approach would be to perform a combination of the PHES, d2 and visuo-motor / bimanual coordination tests. This would identify nearly all patients with some neurological impairment.

The results reported are clinically relevant. It is shown that patients in cluster 3 and, especially, those in cluster 4, who are classified as “without MHE” by PHES, show a high risk of suffering clinical complications, including overt HE. These patients should be followed clinically, as they have a higher risk for progression to overt HE and/or other decompensations compared to patients without MHE with no failed tests.

Most tests proposed here are significantly different from other tests already being used/approved by the guidelines for covert HE. The oral version of the Stroop test used here is similar to the Stroop App already proposed by Bajaj et al (2013) [45]. However, the visuo-motor and bimanual coordination tests, which are very sensitive in detecting MHE, evaluate different functions than the tests being used.

Also the oral SDMT test and, especially, the d2 test evaluate some aspects with more sensitivity than current tests such as the ICT and CDRS tests. Inhibitory Control Tests (ICT) is a computerized test which evaluates attention, working memory, response inhibition, and psychomotor speed [46]. Cognitive Drug Research System (CDRS) is a computerized test that reflects 5 cognitive domains (power and continuity of attention, episodic, working and speed memory) [47].

The Oral version of symbol-digit modalities test (SDMT) allows measuring mental processing speed, without the psychomotor component of digit symbol sub-test from PHES [27].

The d2 test evaluates selective/sustained attention and mental concentration and provides scores reflecting three components of attentional behaviour: speed or amount of work done in a given time; accuracy of such work and relationship between speed and accuracy [25, 26]. This test also measures scanning accuracy and speed and learning and test-taking strategies. Its duration and difficulty allow analysis of the participant’s ability to achieve, shift, and maintain attention (elements of sustained attention); focus on and select target stimuli (elements of selective attention); improve or worsen with practice; and develop strategic approaches to discriminating between targets and non-targets [25].

The combination of PHES with the tests proposed here, especially with d2 and visuo-motor and bimanual coordination tests would significantly increase the number of patients with MHE detected. As shown above, patients in clusters 3 and 4, classified as “without MHE” by PHES but as with MHE with the proposed tests have increased risk of clinical complications and must be diagnosed and clinically followed.

## Conclusions

In summary, the results reported show that the PHES is not sensitive enough to detect the earliest neurological deficits in cirrhotic patients. The use of more sensitive tests such as oral SDMT and bimanual coordination would increase the number of subjects diagnosed of MHE by around 60%. This would allow earliest detection and treatment of MHE and improving quality of life. It is also shown that cirrhotic patients are a heterogeneous population concerning their earliest neurological alterations, likely due to initial alterations in different cerebral mechanisms. Also, patients classified as “without MHE” by PHES belonging to clusters 3 and 4 in our study have a high risk of suffering clinical complications, including overt HE and must be diagnosed and clinically followed. This further supports the need for the use of a combination of sensitive tests to diagnose MHE.
